# Bicuspid Valve Aortopathy: Is It Reasonable to Define a Different Surgical Cutoff Based on Different Aortic Wall Mechanical Properties Compared to Those of the Tricuspid Valve?

**DOI:** 10.3390/jcdd11100312

**Published:** 2024-10-08

**Authors:** Pasquale Totaro, Alessandro Caimi, Giulia Formenton, Martina Musto, Martina Schembri, Simone Morganti, Stefano Pelenghi, Ferdinando Auricchio

**Affiliations:** 1 Division of Cardiac Surgery, IRCCS Foundation Hospital “San Matteo”, 27100 Pavia, Italy; martina.musto01@universitadipavia.it (M.M.); s.pelenghi@smatteo.pv.it (S.P.); 2 Department of Civil Engineering and Architecture (DICAR), University of Pavia, 27100 Pavia, Italy; alessandro.caimi@unipv.it (A.C.); giulia.formenton01@universitadipavia.it (G.F.); martina.schembri01@universitadipavia.it (M.S.); auricchi@unipv.it (F.A.); 3 Department of Electrical, Computer and Biomedical Engineering, University of Pavia, 27100 Pavia, Italy; simone.morganti@unipv.it

**Keywords:** bicuspid aortic valve, ascending aortic aneurysm, mechanical properties

## Abstract

Background: In this study, we examined and compared ex vivo mechanical properties of aortic walls in patients with bicuspid (BAV) and tricuspid (TAV) aortic valve aortopathy to investigate if the anatomical peculiarities in the BAV group are related to an increased frailty of the aortic wall and, therefore, if a different surgical cutoff point for ascending aortic replacement could be reasonable in such patients. Methods: Ultimate stress tests were performed on fresh aortic wall specimens harvested during elective aortic surgery in BAV (n. 33) and TAV (n. 77) patients. Three mechanical parameters were evaluated at the failure point, under both longitudinal and circumferential forces: the peak strain (Pstr), peak stress (PS), and maximum elastic modulus (EM). The relationships between the three mechanical parameters and preoperative characteristics were evaluated, with a special focus on evaluating potential risk factors for severely impaired mechanical properties, cumulatively and comparatively (BAV vs. TAV groups). Results: The patient populations were inhomogeneous, as BAV patients reached surgical indication, according to the maximum aortic dilatation, at a younger age (58 ± 15 vs. 64 ± 13; *p* = 0.0294). The extent of the maximum aortic dilatation was, conversely, similar in the two groups (52 ± 4 vs. 54 ± 7; *p* = 0.2331), as well as the incidences of different phenotypes of aortic dilatation (with the ascending aorta phenotype being the most frequent in 81% and 66% of the BAV and TAV patients, respectively (*p* = 0.1134). Cumulatively, the mechanical properties of the aortic wall were influenced mainly by the orientation of the force applied, as both PS and EM were impaired under longitudinal stress. An age of >66 and a maximum dilatation of >52 mm were shown to predict severe Pstr reduction in the overall population. Comparative analysis revealed a trend of increased mechanical properties in the BAV group, regardless of the position, the force orientation, and the phenotype of the aortic dilatation. Conclusions: BAV aortopathy is not correlated with impaired mechanical properties of the aortic wall as such. Different surgical cutoff points for BAV aortopathy, therefore, seem to be unjustified. An age of >66 and a maximum aortic dilatation of >52 mm, however, seem to significantly influence the mechanical properties of the aortic wall in both groups. These findings, therefore, could suggest the need for more accurate monitoring and evaluation in such conditions.

## 1. Introduction

A bicuspid aortic valve (BAV) is the most common congenital cardiac defect frequently associated with a dilated ascending aorta (with a prevalence of up to 50%) [1,2,3,4,5,6,7]. Two primary hypotheses have emerged regarding the increased prevalence of aortic dilatation in individuals with a bicuspid aortic valve (BAV). The first suggests a genetic predisposition, particularly affecting the aortic root, which contributes to the dilatation [8]. The second hypothesis focuses on the adverse effects of altered blood flow dynamics caused by the structural abnormalities of the valve. These altered flow patterns are especially detrimental to the ascending aorta, where they significantly impact the integrity of the aortic wall [9].

Anatomical, histological, and physiopathological peculiarities in the thoracic aortic walls of BAV patients have been shown [10,11,12,13], thus suggesting a further correlation with an increased risk of acute aortic syndrome, especially aortic dissection [14,15]. In the last decade, several studies, focused on “ex vivo” biomechanical evaluations, have been reported [16,17,18,19,20,21,22,23] to better clarify the physiopathology of aortic diseases in order to identify potential early-predictor risk factors for acute aortic complications. Despite all the confirmed peculiarities of patients with BAV, however, preserved mechanical properties of the aortic wall have been reported compared to patients with a tricuspid aortic valve (TAV) [19,20,21,22,23]. A clear correlation between BAV aortopathy and an increased risk of acute aortic complications related to both aortic rupture [24] and/or aortic delamination [25], leading to aortic dissection, furthermore, have never been demonstrated, and, therefore, no specific indications for surgical treatments have been included in the current guidelines [26,27,28].

Our group, in cooperation with the Department of Engineering at our university, has addressed and reported the “ex vivo” evaluation of the physiological characteristics of aortic walls for many years [29,30,31], and we have also recently reported our findings with respect to the anatomical characteristics in BAV compared to TAV patients [32].

We designed this study in order to analyze, as a primary endpoint, the correlation between preoperative patients’ characteristics and aortic wall mechanical properties, aiming at the identification of “risk factors” for severely impaired mechanical properties to be used in clinical decision-making processes. As a secondary endpoint, we focused on the comparison between mechanical properties in bicuspid valve aortopathy vs. tricuspid valve aortopathy, aiming at the clarification of the potential increased frailty of aortic walls in bicuspid aortopathy.

## 2. Materials and Methods

This prospective study was approved by the Institutional Ethical Committee (n. 20150005619—9 March 2015) to analyze the mechanical properties of aortic walls in patients with a dilated thoracic aorta undergoing elective aortic surgery. In a second step (amendment n. 20200019579—14 February 2020), the study population was also extended to patients undergoing emergency surgery for acute aortic syndrome and patients without aortic disease undergoing heart transplantation (acting as a “control group”). The mechanical test protocol, described below and attached as Appendix A, did not change for the entire duration of the study. Herein, we present our results related to the analysis of uniaxial mechanical tests completed on 110 patients undergoing elective aortic surgery procedures, with or without combined associated procedures, enrolled, following written informed consent, over an 8-year period (April 2016–July 2024). As the purpose of this study was the evaluation and comparison of mechanical properties in patients with a dilated aorta and a native bicuspid or tricuspid aortic valve, data from patients operated on in an emergency and from the control group were not included in this analysis. All the patients were initially referred to our division for aortic dilatation, which was the sole indication for surgery in 74 patients (70%). In contrast, 36 patients (30%) required ascending aorta or aortic root replacement as a part of a combined procedure, following current guidelines, because of a primary indication for aortic valve disease. The study population’s characteristics are summarized as follows in Table 1.

Patients were divided, according to anatomical features of the native aortic valve, into two groups: the tricuspid aortic valve group (TAV, 77 pts, 70%) and the bicuspid aortic valve group (BAV, 33 pts, 30%). Maximum dilatation of the aorta (MaxD), indexed dilatation (ID = maximum diameter/BSA), dilatation area/height ratio (A/Hr) and the different phenotypes of aortic dilatation (AAP = ascending aorta phenotype; RP: root phenotype) were accurately recorded. According to our previously published protocol [29,30,31], the full cylinder of resected aorta was initially divided into two specimens which were stored in fresh isotonic saline solution and immediately sent to the histopathology laboratory of our foundation (for histological analysis) and to the Engineering Department of the University of Pavia for mechanical tests. Anterior (greater curvature) and posterior (lesser curvature) regions of the aorta were identified to facilitate engineering classification of aortic wall specimens.

### 2.1. Mechanical Property Analysis

**Figure 1:**
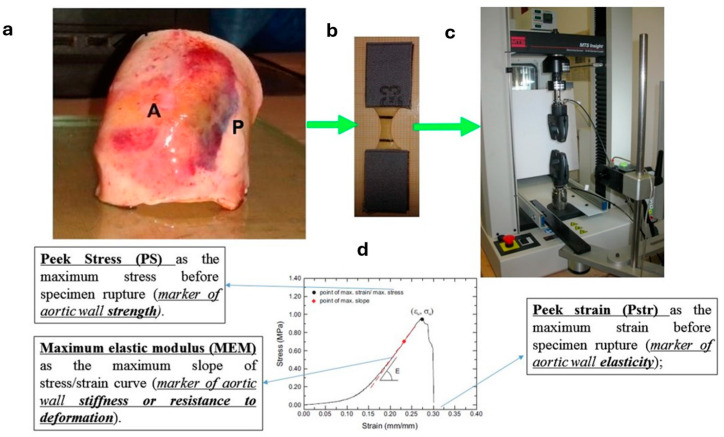
From left to right: (**a**) cylinder of dilated aorta from harvest during ascending aorta replacement; (**b**) specimen from anterior and/or posterior wall prepared for mechanical tests; (**c**) specimen inserted in MTS insight testing system; (**d**) summary of mechanical tests.

Mechanical uniaxial tensile ultimate stress tests were performed on 462 fresh “ex vivo” samples within 24 h of harvest [29,30,31]. Briefly, from the full cylinder (Figure 1a), a number of samples with a dog bone shape and a length/width ratio of at least 4:1 were prepared. Samples were divided and identified according to the region of the aortic wall, as defined in the previous paragraph. The number of samples obtained from each patient were related to the original dimension of the harvested aortic cylinder and ranged from 2 to 13. The dog bone shape (Figure 1b) exhibited a central narrow region, identified by two black markers. Tests were performed using an MTS insight testing system 10 kN (MTS System Corporation, Prairie, MN, USA) using uniaxial circumferential or longitudinal force (Figure 1c). Each test was identified according to the region of the aorta where the specimen was harvested and the direction of the applied force. Three ultimate mechanical property parameters were measured (Figure 1d): Peak Strain (Pstr), as the maximum strain before specimen rupture; Peak Stress (PS), as the maximum stress before specimen rupture; and Maximum Elastic Modulus (EM), as the maximum slope of the stress/strain curve. The full protocol of mechanical tests is attached as Appendix A.

### 2.2. Statistical Analysis

Once tests were completed, the raw data underwent a post-processing phase (to obtain three parameters described above) and then all data were recorded in a designed database. Statistical analysis was performed using Medcalc software (Medcalc 18.2.1; Acacialaan 22, 8400 Ostend, Belgium) in two steps: preliminary results from the first 71 patients enrolled and subsequently on the 110 patients covered by this study. Normal distribution for continuous variables was tested using the Kolmogorov–Smirnov test. Comparative statistics (BAV vs. TAV) was performed, using parametric (unequal variance, two tailed *t*-test) or non-parametric tests (Mann–Whitney for independent samples, Kruskal–Wallis), according the results of the Kolmogorov–Smirnov test, to compare continuous variables of the two study groups. A comparison of mechanical property data according to different categorical variables (i.e., region of aortic wall and direction of the force) was also obtained with a two-way analysis of variance. Spearman’s coefficient of rank correlation (rho) was used to correlate numerical variables to the results of the mechanical test. Multiple logistic regression analysis was used to assess the significant prediction of discrete variables for severely reduced mechanical properties (entering all variables with a *p* < 0.1). For this purpose, severely reduced Pstr, PS and EM were defined according to a value that was <25 percentile (or first quartile). Data were expressed as mean ± sd or median/interquartile range according to a normal distribution.

## 3. Results

### 3.1. Summary of Overall Patient Characteristics

Similarly to our previous report [32], in the pooled analysis, patients in the two groups were not homogeneous in terms of age at surgery. The extents of aortic dilatation (evaluated both in absolute terms and with derived parameters) and the aortic dilatation phenotype were, however, similar in the two groups. The incidence of significant aortic valve disease requiring combined aortic valve surgery was significantly increased in the BAV group. The etiology of aortic valve disease was also significantly different between the two groups, as in the BAV group, aortic stenosis was the predominant cause of valve surgery, while in the TAV group, it was aortic regurgitation (see Table 1).

### 3.2. Cumulative Mechanical Property Analysis (Primary End Point)

**Figure 2:**
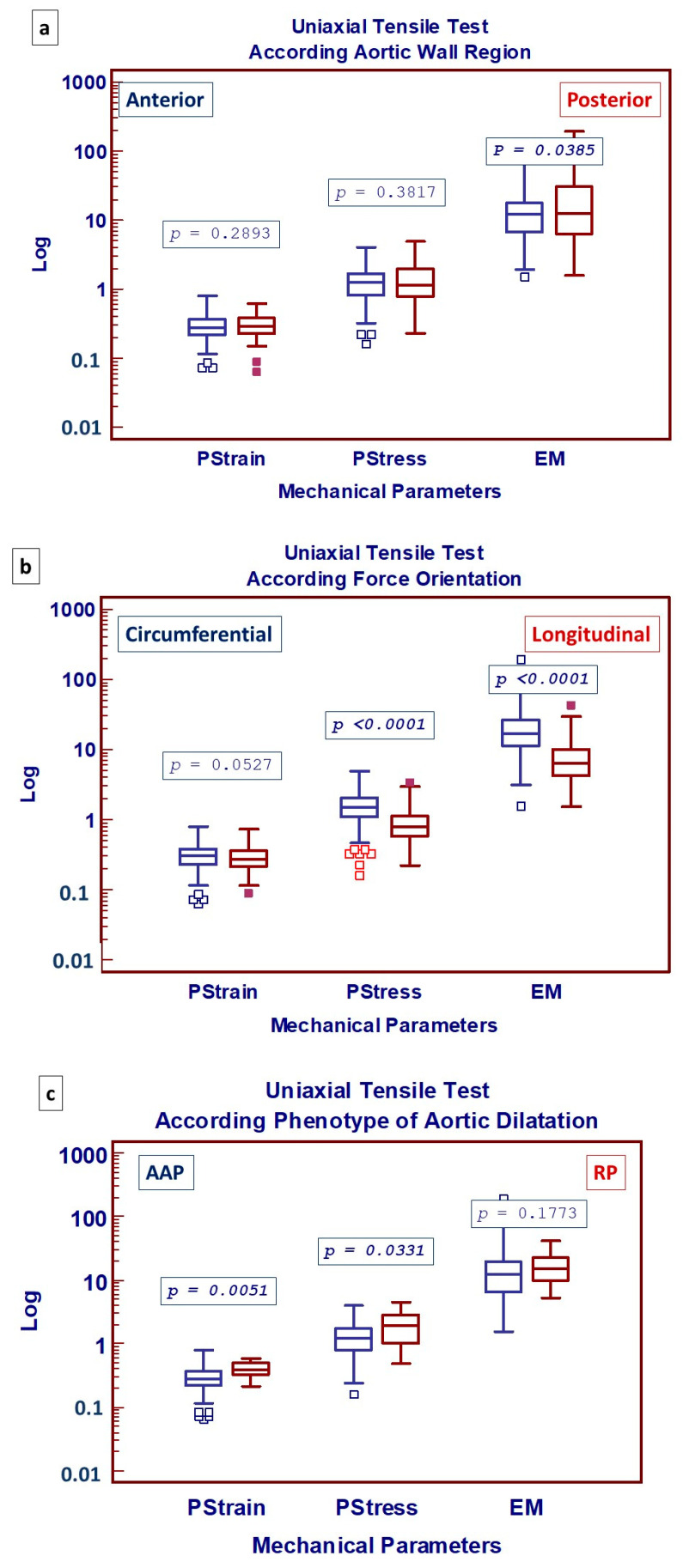
Box-and-whisker dot plots showing comparative analysis of uniaxial mechanical properties with respect to (**a**) the region of aortic wall where specimens were harvested; (**b**) type of force applied; (**c**) phenotype of aortic dilatation: AAP, ascending aorta phenotype; RP, root phenotype; Pstrain, Peak Strain; Pstress, Peak stress; EM, Maximum Elastic Modulus. Borders of box: 1st and 3rd quartile, line in the box: median, whiskers: maximum and minimum values of non-outliers. All values higher/lower than the upper/lower inner/outer fence (3rd/1st quartile ± 1.5/3 IQR) are also plotted as outliers.

The overall analysis of the mechanical properties of the aortic wall, according to the region of the aorta, the direction of the force applied and patient’s phenotype of aortic dilatation is summarized in Figure 2. As shown in Figure 2a, when comparing the mechanical properties of the aortic wall from the anterior (greater curvature) and posterior (lesser curvature) regions of the aorta, only the maximum EM was shown to be significantly increased in the case of the posterior wall specimens. The direction of mechanical traction, on the other hand, did play a significant role in mechanical tests, as both PS and EM were significantly impaired under longitudinal force (Figure 2b). Finally, the ascending aorta phenotype (AAP) of aortic dilatation significantly unfavorably affected both Pstr and PS (Figure 2c).

Looking at the overall correlation between the continuous variable and mechanical properties, Pstr and PS were both inversely correlated to patient age (Spearman’s coefficient of rank correlation (rho) = −0.503—*p* < 0.0001 and −0.118—*p* = 0.0001 for Pstr and PS, respectively). None of the parameters related to the extent of aortic dilatation (maximum diameter, indexed dilatation and area/height ratio) showed, on the other hand, a significant linear correlation with mechanical parameters. Finally multiple logistic regression analysis showed age > 66 and maximum dilatation > 52 mm as predictive of severely impaired Pstr (OR 5.19; 95%CI 2.87 to 9.38; *p* < 0.0001 and OR 3.40; 95%CI 1.73 to 6.68; *p* = 0.0004, respectively).

### 3.3. Comparative (BAV vs. TAV) Mechanical Property Analysis (Secondary End Points)

**Figure 3:**
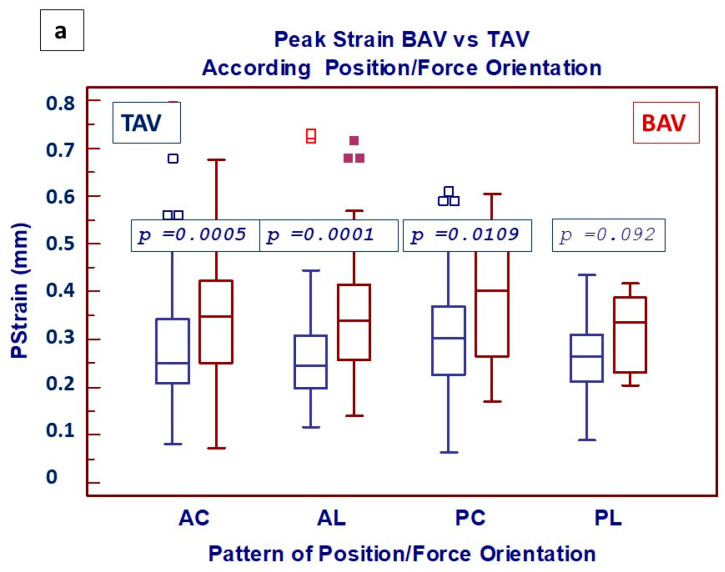

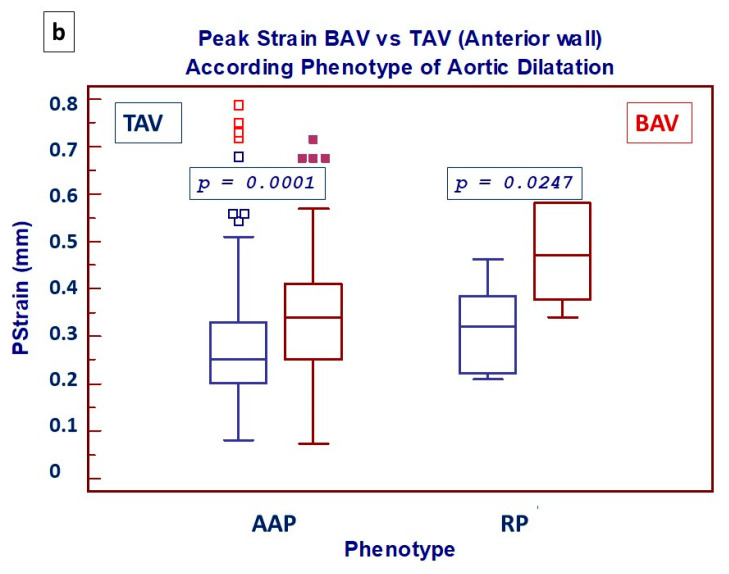
Box-and-whisker dot plots showing (**a**) comparative analysis of Peak Strain between BAV and TAV, according to 4 possible patterns of combination between the region of aorta and the force applied (AC: Anterior/Circumferential; AL: Anterior/Longitudinal; PC: Posterior/Circumferential; PL: Posterior/Longitudinal) and (**b**) specimens from anterior wall, according to the phenotype of aortic dilatation (AAP: ascending aorta phenotype; RP: root phenotype). Borders of box: 1st and 3rd quartile, line in the box: median, whiskers: maximum and minimum values of non-outliers. All values higher/lower than the upper/lower inner/outer fence (3rd/1st quartile ± 1.5/3 IQR) are also plotted as outliers.

Comparative analysis of ex vivo mechanical properties in BAV vs. TAV patients was focused on four different patterns of combination between the position of the aortic wall specimen and the force orientation to include all possible variables. Cumulative Pstr (as shown in Figure 3a) was significantly better preserved in BAV compared to TAV patients, with significant differences in three out of four possible combination patterns (Figure 3a). Looking at the phenotype of aortic dilatation, increased values of Pstr in BAV patients were confirmed (focusing on specimens from the anterior wall), regardless of the type of phenotype considered (Figure 3b).

**Figure 4:**
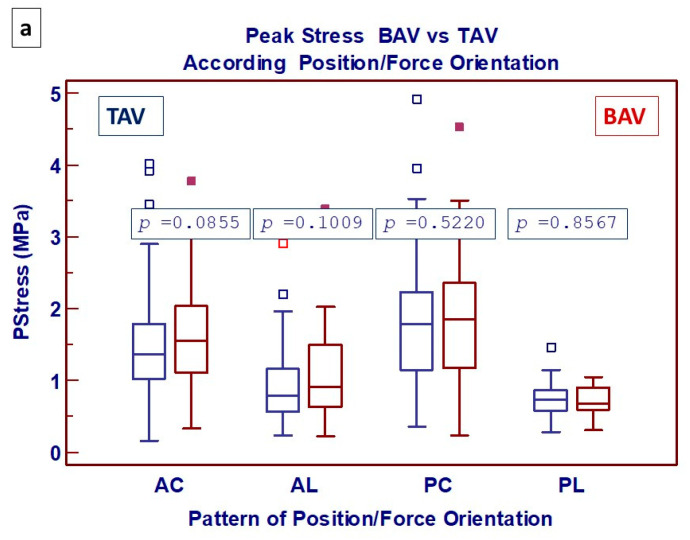

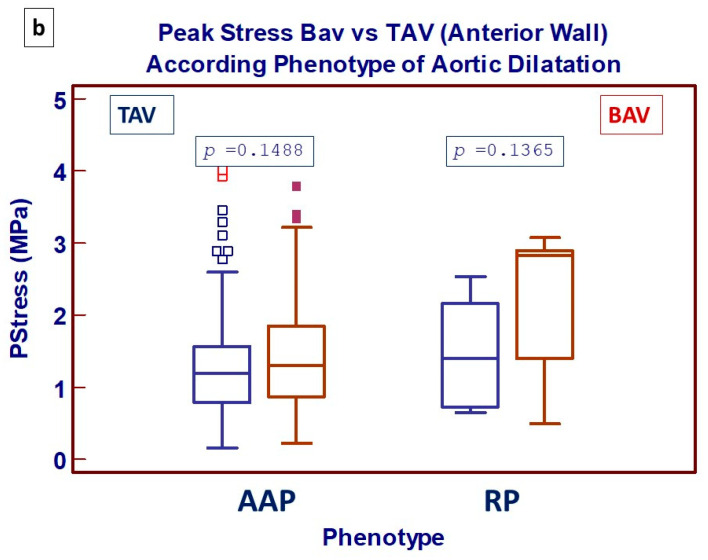
Box-and-whisker dot plots showing (**a**) comparative analysis of Peak Stress between BAV and TAV, according to 4 possible patterns of combination between the region of aorta and the force applied (AC: Anterior/Circumferential; AL: Anterior/Longitudinal; PC: Posterior/Circumferential; PL: Posterior/Longitudinal) and (**b**) specimens from anterior wall, according to the phenotype of aortic dilatation (AAF: ascending aorta phenotype; RF: root phenotype). Borders of box: 1st and 3rd quartile, line in the box: median, whiskers: maximum and minimum values of non-outliers. All values higher/lower than the upper/lower inner/outer fence (3rd/1st quartile ± 1.5/3 IQR) are also plotted as outliers.

PS was also significantly better preserved in BAV specimens in all four combinations of position/orientation but without significant differences (Figure 4a). Looking at the phenotype of aortic dilatation, increased values of PS in BAV patients were, once more, confirmed (focusing on specimens from the anterior wall), regardless of the type of phenotype considered but without reaching statistical significance.

**Figure 5:**
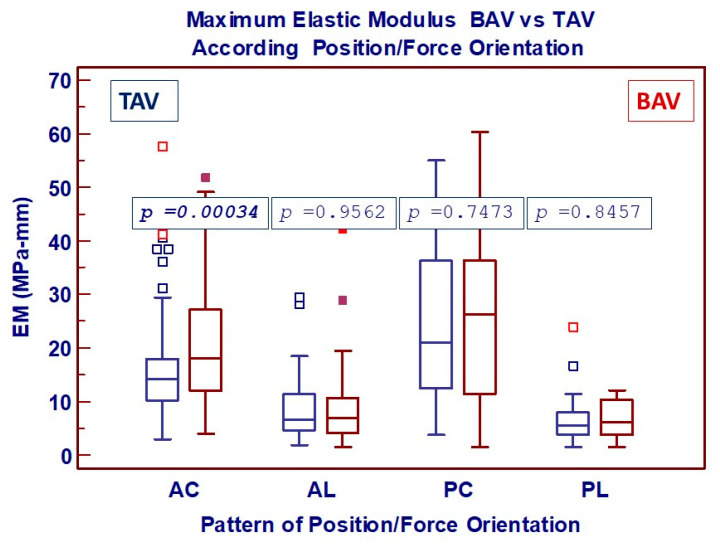
Box-and-whisker dot plots showing comparative analysis of uniaxial mechanical properties (Maximum Elastic Modulus) between BAV and TAV according to 4 possible patterns of combination between the region of aorta and the force applied (AC: Anterior/Circumferential; AL: Anterior/Longitudinal; PC: Posterior/Circumferential; PL: Posterior/Longitudinal) Borders of box: 1st and 3rd quartile, line in the box: median, whiskers: maximum and minimum values of non-outliers. All values higher/lower than the upper/lower inner/outer fence (3rd/1st quartile ± 1.5/3 IQR) are also plotted as outliers.

EM (Figure 5), on the other hand, was significantly different in BAV patients, but only in specimens from the anterior wall under circumferential traction.

**Figure 6:**
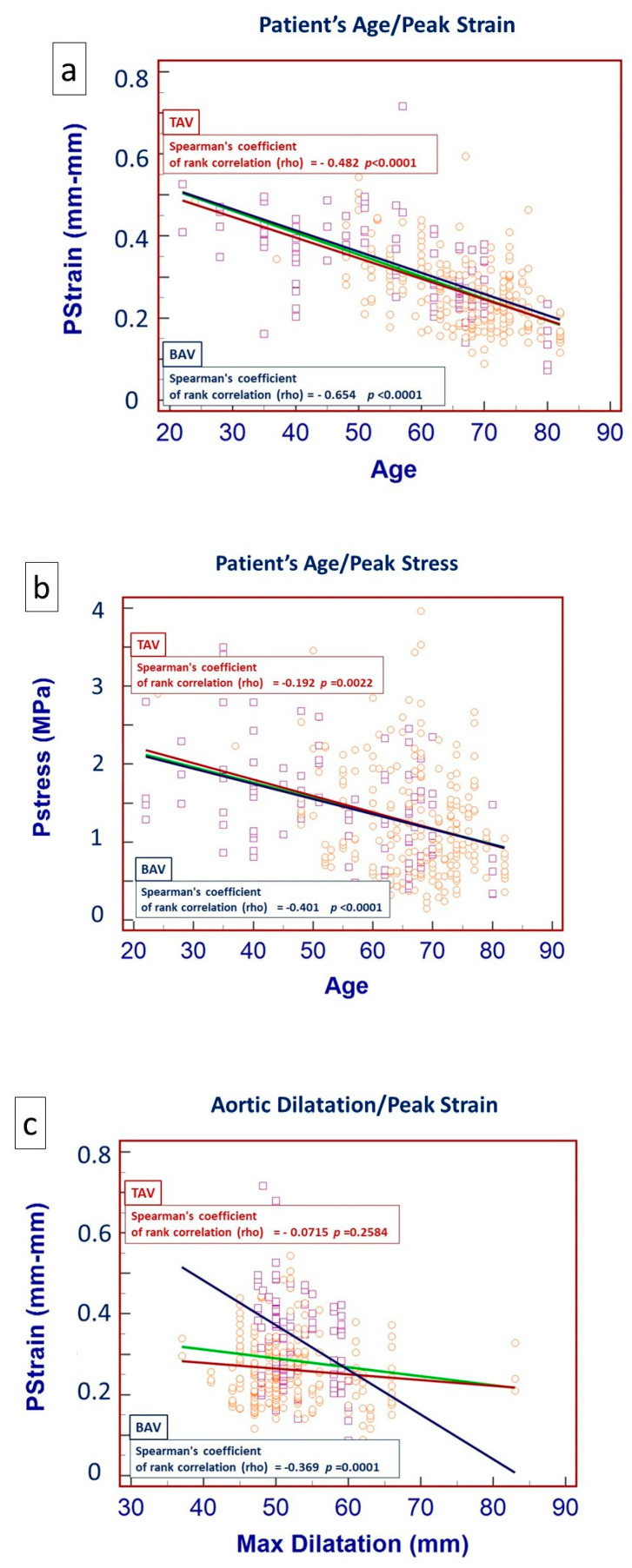
Comparative (BAV vs. TAV) regression line showing the correlation between patient age and peak strain (**a**), patient age and peak stress (**b**) and maximum aortic dilatation and peak strain (**c**).

As far as the linear regression correlation, patient age showed an inverse correlation to PStr (Figure 6a) and PS (Figure 6b) in both BAV and TAV patients, while maximum dilatation showed an inverse correlation to Pstr only in BAV patients (Figure 6c).

Finally, the presence of BAV in multiple logistic regression analysis was shown to be protective against the risk of severely reduced peak strain (OR: 0.47; 95% CI 0.23 to 0.93; *p* = 0.031).

## 4. Discussion

The potential prediction of acute aortic syndromes in patients with aortic dilatation is one of the most debated topics in contemporary medicine. As a matter of fact, for decades, BAV-related aortopathy has been correlated to an increased risk of acute aortic syndromes, especially aortic dissection [15], due to significant anatomical, histological and physiological peculiarity reported in the aortic wall when compared to patients with TAV [10,11,12,13]. However, in 2012, Benedik et al. [25] proved that there were significant differences in aortic wall cohesion between patients with BAV and TAV, suggesting that the major difference was between aortopathy in patients with BAV secondary to aortic valve stenosis and regurgitation [33]. Despite several further studies addressing this issue, evidence of an increased risk of acute aortic syndromes has never been clearly demonstrated, especially in patients with acute aortic dissection with BAV aortopathy [34]. As a consequence, the most recent update of the AHA and ESC guidelines has still not differentiated the inclusion criteria for surgical intervention in the case of ascending aortic aneurysm in BAV patients compared to TAV patients unless additional high-risk conditions arise [26,27,28].

In this study, we focused on a double key issue: overall mechanical testing on the aortic wall of patients with ascending aorta dilatation, undergoing elective surgery, with the aim of detecting significant preoperative risk factors for impaired mechanical properties (primary end point) and comparison between mechanical properties in BAV and TAV patients, aiming to clarify significant differences in aortic wall frailty in BAV patients (secondary end point).

The first interesting piece of evidence in the primary end point was related to the major impact of force orientation on the mechanical properties of the aortic wall, with a significant reduction in aortic wall strength and resistance to the traction under longitudinal stress. The phenotype of aortic dilatation, on the other hand, seems to impact the elasticity and strength of the aortic wall, as they were both reduced in the case of the ascending aorta phenotype. These findings deserve an accurate evaluation considering that if reduced resistance to longitudinal force is well reported [19,20], the increased frailty in the case of the ascending aorta phenotype dilatation seems to be in contrast with a recent report showing an evidently increased risk of acute aortic syndrome in patient with root phenotype aortic dilatation [28]. Analysis of cumulative risk prediction of severely impaired mechanical properties revealed further peculiar and interesting findings, as it showed cutoffs which had either not been considered previously in the current guidelines (patient age: 66 years) or which were significantly below the cutoff reported in the current guidelines for patients without peculiar risk factors (maximum dilatation >52 mm). These findings themselves deserve accurate evaluation and we will discuss this further in the next paragraph related to the potential clinical translation of our results. When we move to the secondary end point of our study, we should stress that our cumulative series of >450 successful tests, with >120 tests from the BAV group, allowed us to carry out a comparative evaluation of the two subgroups, with satisfactory statistical potential, thus overcoming a significant limitation frequently reported in previously published studies. In the comparative analysis focused on the peculiarity of the mechanical properties in the aortic wall in BAV patients compared to TAV patients, we did obtain further interesting findings. In fact, all of the mechanical properties of patients with BAV aortopathy proved to be superior to those of patients with TAV aortopathy. This difference is particularly noticeable in the greater curvature of the aorta, where the dilatation is surely more asymmetrical and where a thinner aortic wall in BAV patients has been previously demonstrated [32]. A higher incidence of aortic stenosis in the BAV group could be correlated with higher post-stenotic dilatation and, therefore, increased asymmetrical wall stress in such patients. The evidence of increased mechanical properties in this scenario surely deserves further investigations. We could speculate that the mechanical differences shown in BAV patients may represent an adaptive response of the aortic wall to the increased wall stress shown in these conditions. It is also important to stress that the peak strain was superior in BAV patients regardless of the force orientation, and therefore even under the longitudinal force orientation, previously identified as a high-stress situation. How do our findings correlate with the current knowledge regarding the ex vivo evaluation of the mechanical properties of the aortic wall? Increased strength of BAV patient samples of the aortic wall represented by the peak stress value is not, by itself, an original finding, as shown in previous smaller series [19,21,22,35,36]. The increased number of fresh (not frozen) samples and the collection from both the anterior and posterior walls, however, represent a significant peculiarity. Our study, furthermore, showed that not only peak stress but also peak strain (which could be considered as a marker of elasticity) was better preserved in specimens from BAV patients. On the other hand, our data related to elastic modulus (indicated in many papers as a marker of the resistance to deformity) in patients with BAV represent an original finding, as it was shown to be reduced in BAV patients in a previous paper [20]. If we try to combine the results from both primary and secondary end points, we could confirm that increased patient age is clearly the main factor correlated with an impairment of mechanical properties (Pstr and PS), both in the BAV group and the TAV group, without a significant difference. We could speculate, however, that patients with BAV usually reach surgical indication at an earlier age [32], and therefore, the age cutoff we showed in this study (66 years) seems to represent a different stage of the natural history of aortic dilatation in BAV patients compared to TAV patients. In other words, the same age cutoff could represent an early phase of disease in many TAV patients, but could represent an advanced phase of disease in many BAV patients, whose aorta usually starts to dilate at a younger age. If we look at the extension of aortic dilatation, it seems that only in the BAV group is there a linear correlation between the extent of the aortic dilatation and the elasticity of the aortic wall. The lack of linear correlation between mechanical properties and maximum diameter, however, was already reported in previous studies [24,37]. However, our study points out that the mechanical properties of the aortic wall (especially peak strain) seem to be significantly impacted by the maximum diameter exceeding 52 mm, regardless of the presence of BAV. In this case, we strengthen what we discussed before, which is the difference in the disease evolution in the two groups, taken into consideration when we consider this parameter.

### Clinical Translation of Ex Vivo Mechanical Tests

Despite the evaluation of “ex vivo” mechanical properties having become a frequent practice [16,17,18,19,20,21,22,23,24,25], the comparison of results from different studies is still problematic due to the lack of standard homogeneous protocols either in test execution or in result analysis. Even more difficult, in our mind, is the attempt to exploit the results of “ex vivo” tests in clinical practice, as previously stressed by other authors [38]. Engineering and the clinical concept of “significant” can indeed be really far from each other, especially in the practical field, such as predicting acute aortic complications in patients with dilated aorta. Intra-patient variability of aortic wall characteristics is evident when more than one sample is collected for each patient [32]. This particular aspect can be approached in different ways, which reflects different engineering and medical attitudes. In the attempt to use the results of mechanical property analysis in clinical practice, we believe that the key point could be identifying the weakest area of the aortic wall, which eventually will be correlated with the development of an acute clinical event. Therefore, in this analysis, we included the results of all accepted tests and we tried to identify potential predictor factors of significantly impaired and mechanical properties of the aortic wall. Unfortunately, there is not a clear cutoff to define severely impaired mechanical properties, and this is the reason why we decided to define severely impaired peak strain, peak stress and maximum elastic modulus by selecting the 25th percentile as the cutoff. Despite this choice, surely deserving further validation, it allowed us to obtain very interesting findings. Based on the results of our comparative analysis of mechanical properties, we could speculate that, despite previously reported differences in anatomical and histological characteristics [27], patients with BAV aortopathy seem to be at a lower risk of acute aortic events when compared to patients with TAV aortopathy, at least when considering a similar extent of dilatation. The protective effect shown by our study is probably more correlated with the mechanism of aortic rupture than with aortic dissection. In our study, we did not carry out specific delamination tests, which, on the other hand, have become quite popular and seem to indicate an increased risk of propagation of aortic dissection in the outer curvature of the aorta [39] and in elderly (>65 yo) female patients [40]. The evidence of preserved mechanical properties in BAV patients could actually suggest the hypothesis of a reduced risk of clinical complication in patients with dilated aorta and BAV. On the other hand, the evidence of a significant impact of maximum diameter > 52 on the mechanical properties of the aortic wall could have a relevant clinical translation, as this cutoff is below the cutoff for surgical indication in current guidelines and could, therefore, suggest the utility of more accurate monitoring of such patients. Our findings related to patient age and mechanical properties are similarly very interesting in terms of clinical implication, since a clear cutoff has never been previously identified and reported, or had less significant clinical impact, despite the aging aorta having been previously correlated with an increased aortic wall thickness [31] and reduced elasticity and strength [24,25,26]. We could speculate that the progressive thickening of the aortic wall reflects a progressive reduction in elasticity and strength of the ascending aorta, which, above 66 years of age, could increase the risk of acute aortic syndrome, once more regardless of the presence of BAV. In conclusion, further studies are crucial in order to standardize the biomechanical test protocol and to validate the translation of mechanical data properties in the clinical scenario. With all the current limits of this type of analysis, our study seems to show, however, that the significant modification of the mechanical properties of the aortic wall in patients with ascending aorta dilatation could start above 66 years of age and 52 mm of dilatation, regardless of the presence of BAV. Patients in such conditions, therefore, should be accurately monitored to assess the potential increased risk of acute aortic syndrome. The accuracy of aortic wall mechanical property prediction based on non-invasive diagnostic tools [41,42] is surely one of the key points to be addressed in future studies.

## 5. Conclusions

Our study shows that the mechanical properties of the aortic wall, when considering similar size of dilatation, are better preserved in BAV patients compared to TAV patients. Furthermore, a maximum dilatation of the aorta > 52 mm and patient age > 66 yo seems to represent a significant risk factor for reduced strength and elasticity in BAV patients compared to TAV patients. Careful monitoring of BAV patients is therefore mandatory, since such cutoffs are below the current surgical indication reported in the current guidelines.

## 6. Limitation

Our study surely carries some limitations. First of all, specimens in this series are all from surgical patients, and therefore, we lack a sort of “control group” with non-diseased aorta. We are trying to address this limitation by extending the study to patients with a normal ascending aorta undergoing a heart transplant. The second limitation of the study is the inhomogeneous patient population between two groups in terms of age and the incidence of aortic valve stenosis. Despite the fact that we acknowledge this potential confounding factor, we decided to include all consecutive patients, as they reflect the natural history of disease in the two groups. We are also addressing this issue by trying to obtain two groups of patients matched for preoperative characteristics. Finally, our study encountered the common limitations of similar studies, represented by the lack of a currently standardized protocol on the execution of mechanical tests and especially on the definition of a significant cutoff for the definition of an abnormal value.

## Figures and Tables

**Table 1:** Overall and subgroup preoperative characteristics. Continuous variables with normal distribution (value expressed as mean ± sd); categorical variables (value expressed as number and % in bracket); BAV = bicuspid aortic valve; TAV = tricuspid aortic valve; BMI = Body Mass Index; BSA = Body Surface Area; AAP = ascending aortic dilatation phenotype; RP = root dilatation phenotype; AVR = aortic valve replacement.

Patients Characteristics	Overall (n.110)	BAV (n.33)	TAV (n.77)	*p*
**Age (years)** **>70**	63 ± 14 39 (35)	58 ± 15 6 (18)	64 ± 13 33 (42)	***0.0294*** ***0.0164***
**Gender** **Male** **Female**	74 (67) 36 (23)	22 (66) 11 (34)	52 (67) 25 (23)	0.898
**Weight (Kg)**	76 ± 15	74 ± 16	76 ± 15	0.409
**Height (m)**	1.70 ± 0.09	1.68 ± 0.09	1.71 ± 0.10	0.156
**BSA (m^2^)**	1.88 ± 0.22	1.85 ± 0.23	1.90 ± 0.22	0.321
**BMI** **>28**	26 ± 4 33 (30)	25 ± 4 6 (18)	26 ± 4 27 (35)	0.837 0.111
**Hypertension**	72 (66)	20 (60)	52 (67)	0.510
**Expected Aortic Diameter (mm)** **Calculated Aortic Ratio**	3.31 ± 0.16 1.60 ± 0.20	3.29 ± 0.17 1.58 ± 0.14	3.32 ± 0.16 1.61 ± 0.23	0.316 0.424
**Max Diameter (mm)** **>50 mm**	53 ± 7 66 (60)	52 ± 4 20 (60)	54 ± 7 46 (59)	0.223 0.924
**Indexed Diameter (mm/m^2^)** **>27.5 mm/m^2^**	28.5 ± 4.7 63 (57)	28.4 ± 3.8 17 (51)	28.5 ± 5.1 46 (59)	0.864 0.528
**Area/Height (cm^2^/m)** **>10 cm^2^/m**	13.2 ± 3.4 99 (90)	12.6 ± 2.0 30 (90)	13.4 ± 3.9 69 (89)	0.287 0.941
**Area/BSA (cm^2^/m^2^)** **>10 cm^2^/m^2^**	12.0 ± 3.5 79 (71)	11.6 ± 2.2 24 (72)	12.2 ± 3.9 55 (71)	0.404 0.981
**AAP Phenotype** **RP Phenotype**	78 (70) 32 (30)	27 (81) 6 (19)	51 (66) 26 (34)	0.113
**Combined AVR** **Aortic Regurgitation** **Aortic Stenosis**	72 (65) 52 (70) 20 (30)	27 (81) 10 (37) 17 (63)	45 (58) 42 (93) 3 (7)	***0.027*** ***0.001***
**Aortic Specimens Characteristics**	**Overall** **(n.462)**	**BAV** **(n.138)**	**TAV** **(n.324)**	***p***
**Anterior Aortic Wall** **Posterior Aortic wall**	313 (68) 149 (32)	103 (74) 35 (26)	210 (64) 114 (36)	0.051

**Figure A1:**
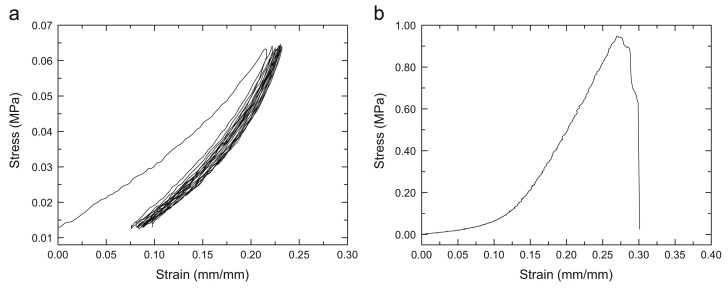
Representative stress–strain curves recorded by the TestWork4 (TWS4): (**a**) preconditioning curve and (**b**) uniaxial tensile test curve.

**Figure A2:**
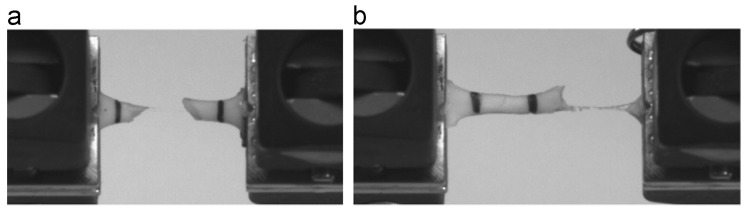
Representative pictures of fractured tissue specimens. (**a**) Rupture occurred inside markers (successful test). (**b**) Rupture occurred outside markers and close to a grip (unsuccessful test).

**Figure A3:**
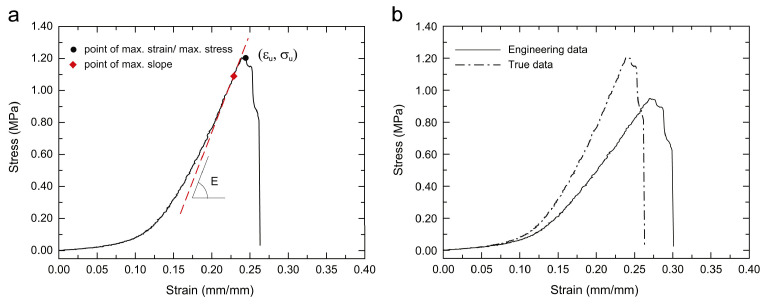
(**a**) Representative stress–strain curve showing the determination of the ultimate mechanical properties (peak strain, peak stress, and maximum elastic modulus). (**b**) Stress–strain curves computed using both engineering and true stress/strain definitions.

## Data Availability

Full database available on specific request.

## Appendix A. [29]

### Appendix A.1. Mechanical Tests

Mechanical tests were performed using the MTS Insight Testing System 10 kN (MTS System Corporation, Prairie, MN, USA) consisting of an electro-mechanical two-column load frame with a moving solid steel cross-head. The MTS system is equipped with a 250 N load-cell (rated force capacity, 250 N) anchored to the cross-head by two pneumatic grips, both suitable for testing soft tissues. The air pressure of the grips was fixed to 20 psi to prevent tissue crushing and, at the same time, specimen slipping. Finally, the specimen extension (i.e., the progressive changes in marker distance) was measured using the ME-46 Video Extensometer (resolution: 1 micron; camera field of view: 200 mm; Messphysik Materials Testing Gmbh, Furstenfeld, Austria).

The mechanical testing procedure was performed in two subsequent steps: (i) preconditioning to stabilize the specimens and to obtain repeatable stress–strain curves, and (ii) uniaxial tensile extension to characterize the mechanical response of the aortic tissue. Preconditioning was achieved by executing 10 consecutive loading–unloading cycles at a constant cross-head speed of 10 mm/min from a minimum load of 0.1 N to a maximum of 0.50 N; see Figure A1a. Uniaxial tensile extension was performed at the same cross-head speed of 10 mm/min until specimen rupture; see Figure A1b. During preconditioning and extension, load and displacement were continuously recorded with a sampling frequency of 10 Hz.

Two representative pictures of fractured specimens are shown in Figure A2, which highlights typical types of specimen rupture occurring during the testing procedure, i.e., either inside the two markers (see Figure A2a) or outside the two markers and close to one of the two grips (see Figure A2b). Only specimens broken inside the two markers were considered as successful tests and included in our study.

### Appendix A.2. Post-Processing

The only experimental data corresponding to successful tests were collected and analyzed in the postprocessing step, which includes data analysis, curve fitting with elastic modulus computation and statistical analysis. In the following, each phase of the post-processing step is detailed.

#### Appendix A.2.1. Data Analysis

Data analysis included the computation of stress, strain and elastic modulus from the quantities directly recorded during the mechanical testing, i.e., the tensile load, *F*, provided by the load cell and the specimen extension, _*d*, measured by the video extensometer.

The load and elongation data were post-processed with Matlab R2011 (The MathWorks, Inc., Natick, MA, USA) to compute the *engineering* stress, *σ_E_*, and the *engineering* strain, *ε_E_*, defined in terms of the initial cross-sectional area and the initial length of the specimen, *A*_0_ and *d*_0_, respectively:

(A1) $$\sigma_{E}=\frac{F}{A_{0}};\;\;\;\;\;\;\;\;\;\varepsilon_{E}=\frac{∆d}{d_{0}}$$

Then, the *true stress*, *σ_T_*, and the *true strain*, *ε_T_*, were computed in terms of the current cross-sectional area, *A*, and the current market distance, *d*, respectively:

(A2) $$\sigma_{T}=\frac{F}{A};\;\;\;\;\;\;\;\;\;\varepsilon_{T}=\int_{d_{0}}^{d}\frac{\delta d}{d}=ln\left(\frac{d}{d_{0}}\right)$$

Assuming material incompressibility, the *true* data can be related to *engineering* data as follows:

(A3) $$\sigma_{T}=\sigma_{E}\left(1+\varepsilon_{E}\right);\;\;\;\;\;\;\;\;\;\varepsilon_{T}=ln\left(1+\varepsilon_{E}\right)$$

By using Equations (A1) and (A3), the *engineering* and *true* stress–strain curves were plotted for each tested specimen; see, for example, Figure 3a. From the stress–strain curve, it is possible to identify the following ultimate mechanical properties (see Figure 3b):

- peak strain, "U, as the maximum strain before specimen rupture;
- peak stress, _U, as the maximum stress before specimen rupture;
- maximum elastic modulus, *E*max, as the maximum slope of the stress–strain curve.

Figure A3 highlights that the ultimate mechanical properties are significantly influenced by the different definitions (*engineering* and *true*) of stress and strain. When the strain is large, the difference between engineering stress and true stress is also very large. In particular, tissue stiffness (i.e., maximum elastic modulus) is underestimated when using engineering measures rather than true ones. Consequently, it is important to use the same definitions for such quantities to properly compare and interpret the obtained results. A comprehensive discussion on the effect of the different stress/strain definitions is presented in our previous study.

#### Appendix A.2.2. Curve Fitting and Elastic Modulus Computation

With the aim of computing the elastic modulus as the derivative of the stress–strain curve, _("), a simple analytic function can be used. According to curve fittings, we obtained the following by using a polynomial function of the seventh order:

(A4) $$\sigma \left(\varepsilon \right)=p_{0}+p_{1}\varepsilon +p_{2}\varepsilon^{2}+p_{3}\varepsilon^{3}+p_{4}\varepsilon^{4}+p_{5}\varepsilon^{5}+p_{6}\varepsilon^{6}+p_{7}\varepsilon^{7}$$

with *p_i_* stress-like coefficients. For each specimen, the coefficients *p_i_* values were obtained by fitting the polynomial function (Equation (A4)) to each experimental stress–strain curve.

Then, the corresponding analytic function for the elastic modulus becomes:

(A5) $$E\left(\varepsilon \right)=\frac{d\sigma }{d\varepsilon }=p_{1}+2p_{2}\varepsilon +{3p}_{3}\varepsilon^{2}+{4p}_{4}\varepsilon^{3}+{5p}_{5}\varepsilon^{4}+6p_{6}\varepsilon^{5}+7p_{7}\varepsilon^{6}$$

From the $E\left(\varepsilon \right)$ set, the maximum elastic modulus was computed.
